# Practices and challenges on coordinating the Brazilian Unified Health System

**DOI:** 10.11606/s1518-8787.2020054001512

**Published:** 2020-02-12

**Authors:** Luzia Beatriz Rodrigues Bastos, Maria Alves Barbosa, Claci Fátima Weirich Rosso, Lizete Malagoni de Almeida Cavalcante Oliveira, Ilma Pastana Ferreira, Diniz Antonio de Sena Bastos, Ana Cláudia Jaime de Paiva, Alex de Assis Santos dos Santos

**Affiliations:** I Universidade Federal de Goiás Faculdade de Enfermagem GoiâniaGO Brasil Universidade Federal de Goiás. Faculdade de Enfermagem. Goiânia, GO, Brasil; II Universidade do Estado do Pará Departamento de Enfermagem BelémPA Brasil Universidade do Estado do Pará. Departamento de Enfermagem. Belém, PA, Brasil; III Universidade do Estado do Pará Departamento de Psicologia BelémPA Brasil Universidade do Estado do Pará. Departamento de Psicologia. Belém, PA, Brasil; IV Universidade Federal de Goiás Faculdade de Enfermagem GoiâniaGO Brasil Universidade Federal de Goiás. Faculdade de Enfermagem. Goiânia, GO, Brasil; V Universidade Federal do Pará Núcleo de Altos Estudos Amazônicos BelémPA Brasil Universidade Federal do Pará. Núcleo de Altos Estudos Amazônicos. Belém, PA, Brasil

**Keywords:** Unified Health System, legislation & jurisprudence, Patient Care Team, organization & administration, Institutional Management Teams, Personnel Management

## Abstract

**OBJECTIVE:**

To analyze the obstacles and challenges faced by managers and coordination professionals in their practices in municipal coordinating centers.

**METHODS:**

An exploratory descriptive study with a qualitative focus, applied in 40 managers and coordination professionals, from September 2017 to November 2018, with semi-structured interviews, resulting in two categories of analysis: limiting factors and factors that facilitate the management and operationalization of the Brazilian Unified Health System (SUS) coordinating sector.

**RESULTS:**

Analyzing the statements, we found evidence of the following limiting factors: failure in the criteria of referral, unavailability of beds, high demand, systemic difficulties in relation to the coordinating system, procedures of difficult scheduling and execution, increased repressed demand for elective procedures and difficulties in the flow of information between primary care and coordination. In the category of facilitating factors, the most significant possibilities were: expansion of the capability to know the user’s reality, improvement in primary care and increase in health financial resources, health training and education and restructuring, in addition to reorganizing internal coordinating procedures.

**CONCLUSION:**

The limiting factors of coordination show the need to promote actions that offer all SUS users full access to health services.

## INTRODUCTION

Regulation is one of the functions of political power, understood as the activity of organizing decision-making processes. The term is discussed in two aspects: first because of the diversity of health systems and scope of the State’s health function; and second by harmonizing sometimes contradictory (economic, social, public or private) interests ^1^ .

On the other hand, the health field has been using the term coordination, associated with the role performed by health systems in general, not only as a function of regulating market relations in health. In the Brazilian public health system, it relates to the State’s activity, linked to the normative, administrative, economic, political and governance functions, used in the different forms of intervention that impose decision measures, searching to achieve optimization in the allocation and distribution of public resources ^2^ .

In the health area, coordinating mechanisms are structured in coordination centers and complexes, which act as a central nervous system between demand and supply in the Unified Health System (SUS), forming its pulsating network, which integrates and articulates devices, including centers of hospitalization, appointments, specialized examinations, elective surgeries, urgency, among others ^3^ . The implementation of coordinating complexes includes a minimum operating structure, composed of furniture and equipment infrastructure, including computer-related equipment; also, it includes a process of permanent training of human resources to prepare local multipliers. Coordinating centers are basic structures of the coordinating complex and act in care areas: urgency, hospitalizations, appointments and specialized and highly complex examinations, among others ^4^ .

Operationalization of work in coordinating structures involves the team of managers and coordinating professionals, who are responsible to ensure the authorization of requests for health procedures. Inserted in the coordinating process, they comply with the hierarchy of care according to the degree of complexity required by the user’s health problem ^5^ .

The manager is responsible for managing all service areas in SUS. He/she is considered the health authority in every sphere of government, whose policy and technique must be guided by the principles of Brazilian sanitary reform. He/she is responsible for conducting health policies and faces tensions that influence the possibility of continuity and consolidation of these policies ^6^^,^^7^ .

Coordinating professionals are links between the elements of the system and users, interacting and facilitating health services accessibility. Their contributions in daily actions include analysis and verification of alternatives presented in each request sent by the primary care professional and the decision for authorizing or not the procedure, considering the need for prioritization.

Brazil is in great need for a coordinating process in health, both in public and private sectors. Without coordination, much of the population needing SUS would roam without health care. Proper coordination by the government is necessary to ensure that decisions remain consistent with the population’s interest.

The study may promote approaches on the work of public management in detecting and overcoming limiting factors of health coordination, suggesting possibilities to achieve a more dignifying access to SUS services by the users, considering that coordination centers are essential in the organizational process of health management, as they constitute areas of reference and articulation, providing a more effective response to requesting units and, above all, to the user. In this sense, it is necessary to investigate whether the municipalities of the state of Pará have traced responsibilities in relation to the coordination of health services and what factors hinder its management within SUS.

This study aims to analyze the obstacles and challenges faced by managers and coordination professionals in their practices in municipal coordination centers.

## METHODS

An exploratory descriptive study with a qualitative focus developed in coordinating centers in four municipalities in Pará. We conducted 40 semi-structured interviews, with 20 health managers (HM) and 20 coordination professionals (CP), corresponding to 45.45% of the total coordination employees. The choice was motivated by the respondents’ availability.

Managers and coordination professionals of higher and secondary education, permanent and temporary, part of the municipal health secretariats, who agreed to participate and consented to the application of the data collection instrument were included in the research. The research excluded coordination professionals from the bed/hospitalization center in Belém, because they performed their work activities at night, being difficult to collect data. Employees of SUS coordination in Pará, for the most part, are women, with an mean age of 39 years and are operating for between two and five years in the coordination. Among the coordination professionals, we find nurses, doctors, dentists, pharmacists and administrative technicians of secondary education.

The study scenario is in the state of Pará, specifically the coordination centers of the municipalities of Belém, Ananindeua, Marituba and Benevides, located in Região Metropolitana I, considered the second most populous metropolitan region in the Northern Region of Brazil, with 2,491,052 inhabitants ^8^ . Belém has an estimated population of 1,485,732 inhabitants and 2,191 health facilities; Ananindeua has 525,566 inhabitants and 238 health facilities; Marituba has an estimated population of 129,321 inhabitants and 45 health facilities; and Benevides has 61,689 inhabitants and 30 health facilities ^8^^,^^9^ . Coordination structures are composed of 11 referenced demand access coordination centers, two in Belém, seven in Ananindeua, one in Marituba, and one in Benevides. They all develop activities of medium and high outpatient and hospital complexity.

Data collection occurred between September 2017 and November 2018, and the collection instrument was a semi-structured interview, applied in work environments, in a reserved area, for approximately 40 minutes. It focused on questions about workers’ experience in coordination, performance, coordination difficulties, coordinating need and user’s access to SUS services. All interviews were recorded and transcribed. We elaborated charts in Microsoft Word, grouping the respondents’ statements, later inserted in the qualitative analysis *software* ATLAS.ti version 8, which provided organization, management and grouping of the textual content. At this stage, data were organized according to their similarities. After coding, the *software* provided a network with the citations of each code, which allowed to categorize the limiting and facilitating factors in the discourse of SUS managers and coordinating professionals.

After the categorization process, we detailed the results and shared all the concrete and subjective meanings of the analyzed narratives. Each code has its meaning, indicated by a set of citations. The citations were classified in a way that could be grouped according to the criteria of pertinence, completeness, homogeneity, exclusivity, and objectivity.

**Figure 1:**
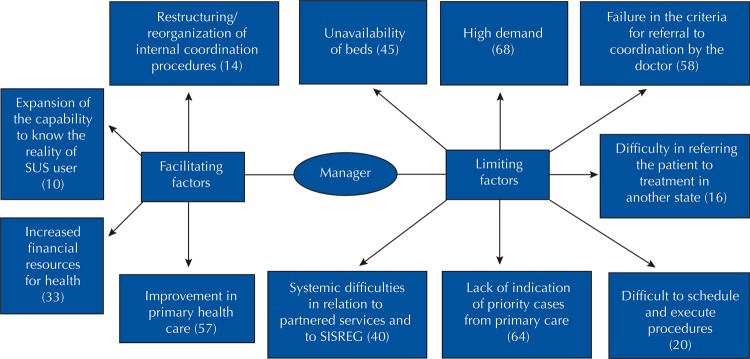
Facilitating and limiting factors evidenced by coordination managers of the Unified Health System of Pará, Brazil, 2018.

SUS: Unified Health System; SISREG: coordination system

**Figure 2:**
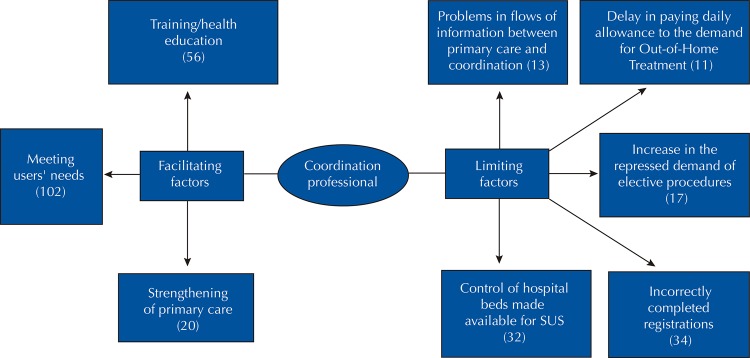
Facilitating and limiting factors evidenced by coordination managers of the Unified Health System of Pará, Brazil, 2018.

SUS: Unified Health System; OHT: out-of-home treatment.

The use of software enabled an organized view of the answers, provided fast and flexible text search engines, allowed establishing keywords that identified text segments, and allowed to link text segments to each other, aiming to establish categories and information networks. It generated a list of the most cited words in the statements of the SUS managers and coordination professionals, which contributed to elaborate two categories of analysis: limiting factors and facilitating factors, present in management and operationalization of SUS coordination sector. Each analysis category generated subcategories, as specified in Figures 1 and 2 . This study was submitted to the Research Ethics Committee of the Hospital de Clínicas of the Universidade Federal de Goiás and approved with CAAE no. 52395815,0,0000,5078, meeting all the committee’s recommendations.

## RESULTS AND DISCUSSION

### Limiting Factors of Coordination

Analyzing the statements, we found evidence of the following limiting factors: failure in referral criteria, unavailability of beds, high demand, systemic difficulties in relation to the coordination system (SISREG), procedures of difficult scheduling and execution, increased repressed demand for elective procedures, and difficulties in the flow of information between primary care and coordination.

#### • Failure in referral criteria

A total of 58 citations indicate gaps in the demands of primary care in terms of documents issued by professionals, which do not meet the protocols instituted in coordination, often incompatible with the user’s need.

“The information coming from the network is insufficient. Sometimes you can barely identify the name and specialty, you do not have clinical information, exams...” (G18)

“Incomplete medical evolution, who does not say the state of the patient in the emergency room.” (G4)

“Disability is sometimes at the base. The doctor could solve it and the patient would leave with a medication; he could leave feeling much better.” (G16)

“[...] many problems of the requesting units: the issue of poorly made registers, poorly filled registers...” (PR15)

“[...] lack of medical test results, which they do not include.” (PR18)

“[...] the poorly made registers. Because of this, the center may deny or return the request.” (PR17)

Incomplete information regarding the clinical status of the primary care user hinders coordinating action, as well as its referral to specialized units, concealing the identification of conditions that could have been solved in the first level of health care. To establish a clinical protocol is crucial for reliable referrals, in addition to providing technical support to the coordination team, which enables the demands from primary care ^10^ .

In privatized systems, predatory logic is constant, requiring coordination to ensure users’ access to SUS services ^11^ . Another aspect refers to the relationship between the municipalities, which has been little cooperative and supportive and has intensified the game in the decision-making arena in the field of partnership of health services. Some municipalities, due to operational difficulties or even to uncompromising partnerships established in the *Programa de Parcerias de Investimentos* (PPI–Investment Partnership Program), fail to comply with the partnership, which causes losses in supplying services to SUS users ^12^ .

Primary care professionals, especially doctors, are held responsible for the failures of referrals. A study highlights that they work under the pressure of the patients’ family members, of colleagues and managers, so that the concept of priority varies according to their own judgment, which often disregards the coordination process and assumes the role of deciding, using personal contacts, telephone calls or contacts by WhatsApp *,* creating an unofficial parallel network ^13^ .

#### • Unavailability of beds

We found 45 citations expressing the difficulty of the coordination sector in ensuring beds for users who are in the emergency rooms of hospitals, in emergency care units (UPAS) or households, in cases of elective procedures.

“I am not even talking about having more hospitals, but bed supply must be reliable. [...] A hospital has 60 beds, but they offer only five in a day, which we know it is not true.” (G2)

“You see the vacant beds, and then no way they are going to question you, because these are SUS’ beds.” (G10)

“When a mother complains that she needs hospitalization, it is difficult for me to see no vacancy in the system.” (G3)

“Coordination is essential for us to be able to serve a patient who needs care and for this patient to go to the right bed.” (PR9)

“[...] make telephone care in the unit, active search for beds by phone, because we supervise the hospital, we care every bed...” (PR15)

“[...] therefore the lack of bed is absurd.” (PR4)

Most hospitalizations occur directly in the hospitals, without intermediation of coordination centers; in some situations, the flow of referral is different, with classification analysis of priority/risk that sometimes reveals problems in accessibility, compromising the continuity and resolvability of care. Services accessibility depends on the location of the supply and the users’ house, which implies transportation, time, distance and travel costs.

Another important aspect concerns the coordination of beds in private hospitals affiliated with SUS, which serve health insurances and private clients, who somehow compete with users of the public health system in the search for beds. In practice, the beds registered in SUS are also used by the private sector for patients of health insurances and private clients ^14^ .

In Belém, some nursing professionals take turns between the hospitalization center and the supervision in hospitals searching for beds. In the other municipalities, nurses do not supervise the hospitals and the situation becomes even more difficult. Most of the time, public hospitals with greater complexity have their beds occupied, and private hospitals affiliated with SUS need to manage their beds between insurance, private, and public patients.

#### • High demand

A total of 68 citations represent the high demand. Some important issues reflect the fragility in SUS accessibility, especially regarding opportunities for the use of health services. Access limitations indicate ineffective coordination practices and dependent on non-formal mechanisms of action, which result in a lack of vacancies and of more complex specialties ^15^ . These ideas are reinforced by the judiciary performance, which directly interferes with the procedures for referral of the coordination centers. Disgruntled users, due to the long wait, go after the Public Prosecutor’s Office (MP) or the Public Defender’s Office to attend their demands, according to reports:

“[...] a demand from the Public Prosecutor’s Office, which has another urgency.” (G1)

“Every week we were in the MP, with problems, prosecution; it is just criticism.” (G5)

“We received demands, from the MP, Defender’s Office, and Ombudsman Office.” (G3)

“Demand is growing and supply is insufficient.” (PR2)

“There is no vacancy for everyone, and this makes us distressed.” (PR7)

“The attorney wanted to know the IDC [International Classification of Diseases], she wanted to know how serious the patient’s disease was.” (PR8)

Noticeably, in the health area, one of the objects of the legal process is the guarantee of care accessibility, mainly hampered by the absence of vacancies. These legal claims demanded by the population may indicate the need to create public policies aimed at breaking the curative logics based on medicalization and hospitalization, as well as a qualified primary care which would alleviate the user’s search for tertiary care. Therefore, we question the technical capacity of the judiciary representatives to intervene in the SUS management ^16^ . It is important to emphasize that the increase in older adults and the active population, without adequate economic insertion, contributes to the increase in violence, bringing relevant consequences in relation to effective responses to health care ^17^ .

#### • Systemic difficulties in relation to the coordination system (SISREG)

Forty citations link to the operationalization of systems, especially SISREG, and to relations with providers related to the partnerships. This is expressed when users require specialized appointments and medical tests that are usually authorized in the coordination, but then rejected by expired quotas and they need to wait until new request and authorization periods are released.

“Our difficulties really are the partnerships. We know the situation in the country, the state and the municipality; some things are being rejected.” (G3)

“Coordination needs a care network working with it.” (G7)

“Today we have problems with laboratories [...].” (G8)

“We could not put together a team that knows how the coordinating system works.” (PR12)

“We must also decentralize SISREG to facilitate.” (PR16)

“Most municipalities refer to Belém, where the largest structure is, the largest network. Most do not know PPI, coordination.” (PR12)

The implementation of SISREG has minimized systemic problems, as it allows municipalities to schedule specialized appointments and medical tests, as well as the request for hospitalizations, in order to enable the demands from primary care, ensuring its impartiality. Nevertheless, the processes of partnership have been unsatisfactory and inconsistent with the population’s real health needs, even with the implementation of SISREG.

The process of services partnership, especially in relation to municipalities with low technological capacity, which demand services of greater complexity, end up overloading others considered larger in terms of possibilities to supply a wider range of services to the population. However, this is not a rule, because private services are concentrated in larger municipalities, which serve health insurances and private clients, sometimes compromising the service to SUS users.

It is important to mention that the number of beds would be enough to meet the need of populations from large municipalities, such as Belém. However, partnership allows residents of municipalities with less case management to move to others to continue their health demands. Without the insertion of all hospital capacity utilization in a coordination center, the partnership commits to ensure the best response to users’ health problems.

#### • Difficult to schedule and–execute procedures

This issue includes computed tomography (CT) scan, magnetic resonance imaging, and urology and orthopedic surgical procedures. In 20 citations, it becomes evident that not all procedures are coordinated, especially when municipalities do not perform them and need to seek care in others. At-risk patients tend to be prioritized.

“Accessibility to specialized appointment is a problem. A survey of potential at-risk patients should be performed to prioritize them.” (G9)

“We will bump into other difficulties, such as the lack of CT scans in the municipality at the moment.” (G11)

“I think the biggest difficulty is just not being able to coordinate all specialties.” (PR14)

“[...] but in the case of hemodialysis, when we see the very large queue, then we know that vacancy is a limited thing.” (PR17)

“We know that this demand only increases, and we need to try to be as fast as possible to enable specialized appointments.” (PR18)

It is crucial to resignify these care system procedures and remodeling to avoid waste of resources and low case management. Regarding magnetic resonance imaging and CT scan, as the performance of medical tests exceeds twice the amount recommended in ministerial parameters, the secondary care network becomes overloaded without depleting primary care. In this sense, it is necessary to articulately and supportively reorder access flows among federal entities.

#### • Increased repressed demand for elective procedures

Seventeen citations evidenced this item.

“We receive more surgical demand for a specialist” (G5)

“We have a very large demand and cannot supply for everyone” (G3)

“[...] it is the lack of beds. We have a very high demand for a reduced number of beds, so we often have to select, which is unfair” (PR7)

“We have to know our priorities, because we have a very high demand for elective patients.” (PR8)

Elective procedure is all care provided to the user in a surgical environment, with established diagnosis and indication of surgery to be performed with the possibility of prior scheduling, without urgency or emergency character, and should occur within the time limit of 180 days. A study reveals that the demand suppressed by elective surgery in Brazil is mainly caused by a gap in the table of values for SUS procedures, which does not cover the actual care values. The exhausting waiting time for elective surgeries is a management problem at different levels of complexity, both due to the inability to meet demand and to the poor integration of care networks ^18^ .

Several aspects reinforce that coordination centers hardly contribute to impact users’ access to procedures of medium complexity of municipal responsibility. Thus, the centralizing action of state management can be attributed to this issue, which often hinders municipal autonomy ^19^ .

Notably, in the reports, sometimes the coordinating process must be maintained to ensure care, sometimes it must be shut down by hindering flows and bureaucratizing the health system. The decision must be made and assumed to ensure the health services accessibility.

#### • Problems in the flow of information between primary care and coordination

This item was found in 13 citations, as exemplified below:

“[...] this makes the center reject or return the request so that new information is added.” (G6)

“We need to know the tools, the systems that are being worked ...” (G9)

“Now I see a great lack of information, by the doctor, the nurse [...]” (PR1)

“The issue of communication, the immediate return, even if it is by phone, by email, we need information and to always communicate with them, and they with us.” (PR7)

The lack of communication between the services and/or professionals that make up primary care and the specialized care of the municipality exercised in the coordination centers denotes care network vulnerability ^20^ . The integration between primary and specialized care must be a strategic measure to face various challenges in the coordination sector. Indirect challenges (political level) can be faced if coordination presents considerable advances in direct challenges (technical level). This requires skills in planning and executing a strategic plan to subsidize more accurately the political level ^21^ .

## Facilitating Factors of Coordination

The most recurrent and significant possibilities expressed in the managers’ narratives were: expanding the possibility to know the reality of SUS users, improving primary care and increasing financial resources for health, training and health education, and restructuring and reorganizing internal coordination procedures.

### • Expansion of the capability to know the reality of SUS user

This need is expressed in 10 narratives.

“The coordination must know the entire health extension network, see how to offer services to our users based on the demand for primary care.” (G5)

“They have several basic pathologies; they are in constant danger to have a heart attack. We need to hospitalize them, so we have to try to support them.” (G10)

“[...] we would be able to better analyze the patient’s real clinical picture and we would really be able to triage in the right way.” (PR14)

“We try to verify the patient’s profile to directly refer him to the hospital that meets his needs.” (PR18)

Public coordination instruments such as financing, service provider network, registers of provider units, care programming, computerized coordination centers and monitoring of health care actions, among others, are important and are challenges in implementing public coordination. This reinforces the need to expand coordinating mechanisms, as SUS has limited resources and criteria are essential to prioritize user’s access, supplying health actions and services proportional to the different needs ^22^ .

### • Improvement in primary care and increases in financial resources for health

This theme receives 57 citations, with reports related to the lack of specialists in the primary care network, as well as in the basic gynecology and pediatric clinics, to low case management, which affects referrals considered unnecessary to coordination, and to the responsibility and commitment to serving users with a more appropriate response to their health problems.

“Primary care has to serve the patient, to be closer, to be more responsible for him.” (G2)

“To improve work, they should improve the base.” (G4)

“Case management has to be in the basic health unit.” (G5)

“I think if you improve primary care, the patient will not need to go to the specialized care.” (G12)

“If primary care improves, a lot will improve.” (PR8)

“[...] primary care could handle 90% of cases.” (PR12)

“There should be case management in primary care.” (PR13)

“It is important to strengthen primary care because, if it does not, the flow will not decrease.” (PR14)

Increases in financial resources are in 33 narratives:

“I think they should pay more attention to health finance.” (G5)

“To service really improve, I think municipalities need to discuss again the integrated partnered schedule.” (G6)

“The system is unified, all principles and guidelines are written, but this financial compensation is necessary.” (PR14)

“I think if a bigger resource to hire doctors was offered, to reinforce the base [...].” (PR17)

Among the ministerial policies to encourage primary care, the *Programa de Melhoria do Acesso e da Qualidade da Atenção Básica* (PMAQ-AB–National program for access and quality improvement in primary care) is notable, which focuses on achieving its objective with a quality standard guarantee in order to ensure greater transparency and effectiveness of government actions directed to this level of care. It comprises four phases: adherence and initial coordinating care contract, development, external evaluation, and coordinating care recontract ^23^ . In its main objectives, PMAQ proposes changes in the managers and primary care professionals’ actions, to promote access and quality in the health care network ^24^ .

A set of elements which base the development of health care networks deserves highlight: the communicator center (primary health care), secondary and tertiary care spots (specialized services), support systems (diagnostics and therapeutics, among others), logistics systems (user card and others), and governance system ^25^ .

Alternatives to reconstruct and value primary care include: *Requalifica UBS* (Requalification Program of Basic Health Units), creation of the new *Sistema de Informação da Atenção Básica* (SIAB–Primary Care Information System), *Estratégia e-SUS Atenção Básica* (e-SUS Primary Care Strategy), *Programa Telessaúde Brasil Redes* (Telehealth Network Program), PMAQ-AB, and *Política Nacional de Atenção Básica* (National Primary Care Policy), restructuring of the *Programa Saúde na Escola* (PSE–Health at School Program), *Programa Academia da Saúde* (PAS–Health Academy Program), *Política Nacional de Alimentação e Nutrição* (PNAN–National Food and Nutrition Policy), *Política Nacional de Educação Permanente em Saúde* (PNEPS–National Policy on Permanent Health Education) for primary care professionals, *Plano Nacional de Educação Médica* (National Medical Education Plan), *Programa de Valorização dos Profissionais na Atenção Básica* (PROVAB–Primary Care Professional Valorization), and *Programa Mais Médicos* (More Doctors Program) ^25^ . It is noteworthy that several changes are occurring after the new presidential government in 2019, and the permanence of aforementioned strategies to reconstruct and valorize primary care are unsure.

### • Health education and training

The need for health education was expressed in 56 citations.

“[...] sometimes we do not have a defined action for many situations occurring here, sometimes we just have to guess, there is nothing written to me: ‘You have to use this tool.’” (PR9)

“I think we should have more training, more protocols. I think protocols are fundamental because they support the professional’s doing.” (PR6)

“We have to know our priorities, because we have a very high demand for elective patients.” (PR22)

“We have to work multidisciplinarily. [...] I ask: ‘Are you an ICU patient? What are the test results? Do you have tests? Do you have a CT scan? Do you have a magnetic resonance imaging?’” (PR20)

Health institutions need to implement permanent education projects, in due course articulated with human resources–training institutions. Such projects can occur in the workspace, to develop skills focused on improvements and with the participation of all actors involved, based on the horizontal knowledge construction and in an interdisciplinary way. Mostly, continuing health education allows training professionals from the perspective of the expanded concept of health, based on SUS principles and guidelines, emphasizing social control ^26^ .

### • Restructuring and reorganization of internal coordination procedures

Fourteen citations expressed this item.

“You cannot do anything but being organized. I see coordination as the heart of a whole management.” (G3)

“Above all, it is about ensuring the coordination structuring, enabling this structure to receive demands, both from capital and countryside, and decentralizing access to appointments and partnered tests.” (G8)

“With coordination, we can control the beds available to the public network, the user’s care. I think it is of fundamental importance in organizing the service.” (G9)

The success of coordination centers, which intend to be protagonists in the process, depends on collective construction, and their strengthening points consist in establishing partnerships with providers.

In a study conducted in Diadema (SP), named “Facing medical doctors: a communicative management strategy to qualify ambulatory access regulation”, physicians point out their perception as coordinators and see the outpatient coordination center as an important observatory of the health care network, capable of producing information to support decision-making in management ^27^ . To restructure coordination procedures is important to ensure access and equity, and to make the health network manageable and humanized, constituting a power to encourage evaluation for decision-making.

## CONCLUSION

Detailing the limiting factors of coordination, expressed in the interviewees’ statements, offers an overview of the obstacles faced by health coordinators and professionals. It demonstrates the need to raise voices and join forces in actions that address everyone in health services accessibility. These actions should begin by expanding the capability to know the user’s reality and by implementing ministerial policies that enable the improvement of primary care and the increase of financial resources, that enable training and education based on continuing health education and that allow the restructuring and reorganization of internal coordinating procedures, ensuring access and equity of services, supported by health resolution and humanizing practices.

In response to limiting factors, we recommend: a) improvement in the organization of care flow at all levels of the health care network, to impact the most vulnerable health indicators; b) guaranteeing of beds by the intermediation of health coordination centers; c) policies that encourage dehospitalization and ensure the effectiveness of primary health care; d) qualification of workers in the use of various systems, including SISREG; and e) integration between primary and specialized care.
