# Supplementary material for: Practices and challenges on coordinating the Brazilian Unified Health System
Source: Rev Saude Publica. 2020 Feb 12;54:25. doi: 10.11606/s1518-8787.2020054001512 (PMC7017982; doi:10.11606/s1518-8787.2020054001512)
Supplement: Translation

# Translation: Práticas e desafios da regulação do Sistema Único de Saúde

**Authors:** Luzia Beatriz Rodrigues Bastos, Maria Alves Barbosa, Claci Fátima Weirich Rosso, Lizete Malagoni de Almeida Cavalcante Oliveira, Ilma Pastana Ferreira, Diniz Antonio de Sena Bastos, Ana Cláudia Jaime de Paiva, Alex de Assis Santos dos Santos

**Affiliations:** I Brasil Universidade Federal de Goiás. Faculdade de Enfermagem. Goiânia, GO, Brasil; II Brasil Universidade do Estado do Pará. Departamento de Enfermagem. Belém, PA, Brasil; III Brasil Universidade do Estado do Pará. Departamento de Psicologia. Belém, PA, Brasil; IV Brasil Universidade Federal de Goiás. Faculdade de Enfermagem. Goiânia, GO, Brasil; V Brasil Universidade Federal do Pará. Núcleo de Altos Estudos Amazônicos. Belém, PA, Brasil

**Keywords:** Sistema Único de Saúde, legislação & jurisprudência, Equipe de Assistência ao Paciente, organização & administração, Equipes de Administração Institucional, Administração de Recursos Humanos

## Abstract

**OBJETIVO:**

Analisar os entraves e desafios enfrentados pelos gestores e profissionais de regulação em suas práticas nas centrais reguladoras municipais.

**MÉTODOS:**

Estudo descritivo exploratório com enfoque qualitativo, aplicado em quarenta gestores e profissionais de regulação, no período de setembro de 2017 e novembro de 2018, por meio de entrevista semiestruturada, resultando em duas categorias de análise: fatores limitantes e fatores facilitadores da gestão e operacionalização do setor de regulação do SUS.

**RESULTADOS:**

Na análise dos enunciados, foram encontradas evidências dos seguintes fatores limitantes: falha nos critérios de encaminhamento, indisponibilidade de leitos, grande demanda, dificuldades sistêmicas em relação ao sistema de regulação, procedimentos de difícil agendamento e execução, aumento da demanda reprimida de procedimentos eletivos e dificuldades no fluxo de informações entre a atenção primária e a regulação. Na categoria de fatores facilitadores, as possibilidades mais significativas foram: ampliação da capacidade de conhecer a realidade do usuário, melhoria na atenção primária e incrementos de recursos financeiros para a saúde, capacitação e formação em saúde e reestruturação, além de reorganização dos procedimentos internos de regulação.

**CONCLUSÃO:**

Os fatores limitantes da regulação demonstram a necessidade de fomentar ações que ofereçam a todos os usuários do SUS o acesso pleno aos serviços de saúde.

## INTRODUÇÃO

A regulação é uma das funções do poder político, entendida como a atividade de organização dos processos decisórios. Discute-se o termo em duas vertentes: primeiro por ser um conceito polissêmico, pela diversidade dos sistemas de saúde e abrangência da função do Estado na saúde; e segundo por harmonizar interesses por vezes contraditórios (econômicos, sociais, públicos ou privados) ^1^ .

O termo regulação tem sido utilizado no âmbito da saúde, associado à função desempenhada pelos sistemas de saúde em geral, não sendo apenas uma função de regulação das relações de mercado na saúde. No sistema público de saúde brasileiro, relaciona-se à atividade do Estado, ligada às funções normativa, administrativa, econômica, política e de governança, utilizadas nas diferentes formas de intervenção que impõem medidas nas esferas de decisão, na busca por alcançar a otimização na alocação e na distribuição dos recursos públicos ^2^ .

Na área da saúde, os mecanismos regulatórios são estruturados em complexos e centrais de regulação, que atuam como centro nervoso entre demanda e oferta no Sistema Único de Saúde (SUS), formando sua rede pulsante, que integra e articula dispositivos, entre eles as centrais de internação, consultas, exames especializados, cirurgias eletivas, urgência, entre outras ^3^ . A implantação dos complexos reguladores contempla uma estrutura mínima de funcionamento, composta por infraestrutura de mobiliário e equipamentos, inclusive de informática; além de um processo de capacitação permanente de recursos humanos, para preparação de multiplicadores locais. As centrais de regulação são estruturas básicas do complexo regulador e atuam em áreas assistenciais: urgência, internações, consultas e exames especializados, alta complexidade, entre outras ^4^ .

A operacionalização do trabalho nas estruturas de regulação envolve a equipe de gestores e profissionais reguladores, que têm a responsabilidade de assegurar a autorização das solicitações de procedimentos de saúde. Inseridos no processo regulatório, obedecem à hierarquização do atendimento de acordo com o grau de complexidade exigido pelo problema de saúde do usuário ^5^ .

O gestor é responsável por administrar todas as áreas de prestação de serviços no SUS. É considerado a autoridade sanitária em cada esfera de governo, cuja política e técnica deve estar pautada pelos princípios da reforma sanitária brasileira. Tem a responsabilidade de conduzir as políticas de saúde e enfrenta tensões que influenciam a possibilidade de continuidade e consolidação dessas políticas ^6^^,^^7^ .

Os profissionais reguladores se colocam na posição de elos entre os elementos do sistema e os usuários, interagindo e facilitando o acesso aos serviços de saúde, suas contribuições em ações cotidianas incluem análise e verificação de alternativas apresentadas em cada solicitação enviada pelo profissional da atenção primária e a decisão pela autorização ou não do procedimento, considerando a necessidade de priorização.

No Brasil, há grande necessidade do processo regulatório na saúde, tanto no setor público quanto nos privados. Sem regulação, grande parte da população que necessita do SUS ficaria peregrinando sem atendimento de saúde. É necessária uma regulação adequada por parte do poder público para garantir que as decisões permaneçam consistentes com o interesse da população.

O estudo poderá fomentar abordagens sobre o papel da gestão pública na detecção e superação de fatores limitantes da regulação em saúde sugerindo possibilidades para o alcance do acesso mais digno de usuários aos serviços do SUS, considerando que as centrais de regulação são determinantes no processo organizacional da gestão da saúde, pois constituem áreas de referência e articulação, fornecendo resposta mais efetiva às unidades solicitantes e, sobretudo, ao usuário. Nesse sentido, torna-se necessário investigar se os municípios do estado do Pará traçaram responsabilidades em relação à regulação dos serviços de saúde e quais fatores dificultam a gestão dessa regulação no âmbito do SUS.

O objetivo do estudo é analisar os entraves e desafios enfrentados pelos gestores e profissionais de regulação em suas práticas nas centrais reguladoras municipais.

## MÉTODOS

Estudo descritivo exploratório com enfoque qualitativo desenvolvido em centrais reguladoras de quatro municípios paraenses. Foram realizadas 40 entrevistas semiestruturadas, sendo vinte gestores (G) e vinte profissionais de regulação (PR), correspondendo a 45.45% do total de funcionários de regulação. A escolha foi motivada em razão da disponibilidade dos pesquisados.

Foram incluídos na pesquisa gestores e profissionais de regulação de nível superior e médio, efetivos e temporários, componentes do quadro das secretarias municipais de saúde, que aceitaram participar e consentiram na aplicação do instrumento de coleta de dados. A pesquisa excluiu profissionais de regulação da central de leitos/internações de Belém, pelo fato de desempenharem suas atividades laborais no horário noturno, constituindo uma dificuldade de operacionalização da coleta de dados. Os funcionários da regulação do SUS no Pará, em sua maioria, são do sexo feminino, com idade média de 39 anos e entre dois e cinco anos de atuação na regulação. Entre os profissionais de regulação, destacam-se enfermeiros, médicos, dentistas, farmacêuticos e técnicos administrativos de nível médio.

O cenário do estudo é no estado do Pará, especificamente as centrais de regulação dos municípios de Belém, Ananindeua, Marituba e Benevides, situados na Região Metropolitana I, considerada a segunda região metropolitana mais populosa da Região Norte do Brasil, com 2.491.052 habitantes ^8^ . Belém tem uma população estimada em 1.485.732 habitantes e 2.191 estabelecimentos de saúde, Ananindeua apresenta 525.566 habitantes e 238 estabelecimentos de saúde, Marituba apresenta uma população estimada em 129.321 habitantes e 45 estabelecimentos de saúde e em Benevides estima-se que haja 61.689 habitantes e 30 estabelecimentos de saúde ^8^^,^^9^ . As estruturas de regulação estão compostas por 11 centrais de regulação do acesso de demanda referenciada, sendo duas em Belém, sete em Ananindeua, uma em Marituba e uma em Benevides. Todas desenvolvem atividades de média e alta complexidade ambulatorial e hospitalar.

A coleta de dados ocorreu entre os meses de setembro de 2017 e novembro de 2018, e o instrumento de coleta foi uma entrevista semiestruturada, aplicada nos ambientes de trabalho, em área reservada, com duração média de 40 minutos. Ela pautou-se em questões sobre experiência dos trabalhadores na regulação, desempenho das ações, dificuldades na regulação, necessidade regulatória e acesso dos usuários aos serviços do SUS. Todas as entrevistas foram gravadas e depois transcritas. Foram elaborados quadros no Microsoft Word agrupando as falas dos pesquisados, posteriormente inseridos no *software* de análise qualitativa ATLAS.ti versão 8, que proporcionou a organização, gerenciamento e agrupamento de conteúdo textual. Nessa etapa os dados foram organizados de acordo com suas semelhanças. Após a codificação, o *software* permitiu gerar uma rede com as citações de cada código, o que permitiu categorizar os fatores limitantes e facilitadores apresentados no discurso dos gestores e profissionais reguladores do SUS.

Após o processo de categorização, foram detalhados os resultados obtidos e compartilhados todos os significados concretos e subjetivos das narrativas analisadas. Cada código tem seu significado, indicado por um conjunto de citações. As citações foram classificadas de forma que pudessem ser agrupadas obedecendo aos critérios de pertinência, exaustividade, homogeneidade, exclusividade e objetividade.

O uso do *software* possibilitou uma visão organizada das respostas, proporcionou mecanismos de busca de texto rápida e flexível, permitiu estabelecer palavras-chave que identificaram segmentos de texto e possibilitou atrelar segmentos de texto uns aos outros, com o objetivo de estabelecer categorias e redes de informação. Ele gerou uma lista de palavras mais citadas nos enunciados dos gestores e profissionais de regulação do SUS, que contribuíram para a elaboração de duas categorias de análise: fatores limitantes e fatores facilitadores apresentados na gestão e operacionalização do setor de regulação do SUS. Cada categoria de análise gerou subcategorias, conforme especificado nas Figuras 1 e 2 . O presente estudo foi submetido ao Comitê de Ética em Pesquisa do Hospital de Clínicas da Universidade Federal de Goiás e aprovado com o CAAE nº 52395815.0.0000.5078, atendendo a todas as recomendações do comitê.

**Figura 1:** Fatores facilitadores e limitantes evidenciados pelos gestores de regulação do Sistema Único de Saúde do Pará, 2018.

SUS: Sistema Único de Saúde; SISREG: sistema de regulação

**Figura 2:** Fatores facilitadores e limitantes evidenciados pelos profissionais de regulação do Sistema Único de Saúde do Pará, 2018.

SUS: Sistema Único de Saúde; TFD: tratamento fora do domicílio.

## RESULTADOS E DISCUSSÃO

### Fatores Limitantes da Regulação

Na análise dos enunciados, foram encontradas evidências de sete subcategorias de fatores limitantes: falha nos critérios de encaminhamento, indisponibilidade de leitos, grande demanda, dificuldades sistêmicas em relação ao sistema de regulação (SISREG), procedimentos de difícil agendamento e execução, aumento da demanda reprimida de procedimentos eletivos e problemas no fluxo de informações entre a atenção primária e a regulação.

#### • Falha nos critérios de encaminhamento

São apontadas 58 citações que indicam as lacunas nas demandas da atenção primária em termos de documentos emitidos pelos profissionais, que não atendem aos protocolos instituídos na regulação, muitas vezes incompatíveis com a necessidade do usuário.

“As informações que vêm da rede são insuficientes. Às vezes você mal consegue identificar o nome e a especialidade, não tem informações clínicas, exames…” (G18)

“Evolução médica incompleta, que não diz o quadro daquele paciente que está lá no pronto-socorro.” (G4)

“A deficiência às vezes está lá na ponta. O médico poderia resolver e o paciente sair dali com uma medicação, poderia sair dali bem melhor.” (G16)

“[…] muitos problemas assim das unidades solicitantes: a questão de cadastros mal feitos, mal preenchidos…” (PR15)

“[…] falta de resultado de exames, que eles não colocam.” (PR18)

“[…] são os cadastros mal feitos. Isso acaba fazendo com que a central negue ou devolva a solicitação.” (PR17)

Informações incompletas em relação ao estado clínico do usuário referenciado da atenção primária dificultam a ação regulatória, bem como seu encaminhamento para unidades especializadas, escamoteando a identificação de condições que poderiam ter sido solucionadas no primeiro nível de atenção à saúde. O estabelecimento de um protocolo clínico é crucial para encaminhamentos fidedignos, além de proporcionar apoio técnico à equipe de regulação, que viabiliza as demandas provenientes da atenção primária ^10^ .

Nos sistemas privatizados a lógica predatória é uma constante, sendo necessário o componente regulador para garantir o acesso dos usuários aos serviços do SUS ^11^ . Outro aspecto se refere à relação entre os municípios, que tem se mostrado pouco cooperativa e solidária e tem acirrado o jogo na arena decisória no campo da pactuação de serviços de saúde. Alguns municípios, por dificuldades operacionais ou até mesmo por descompromisso dos acordos estabelecidos na PPI, deixam de cumprir o acordado, o que acarreta prejuízos na oferta de serviços aos usuários do SUS ^12^ .

Os profissionais da atenção primária, especialmente os médicos, são responsabilizados pelas falhas de encaminhamentos. Um estudo destaca que eles trabalham sob a pressão dos familiares de pacientes, dos colegas e dos gestores, de modo que o conceito de prioridade varia conforme seu próprio julgamento, que muitas vezes desconsidera o processo regulatório e toma para si o papel de decidir, se utilizando de contatos pessoais, ligações telefônicas ou contatos pelo WhatsApp, criando uma rede paralela não oficial ^13^ .

#### • Indisponibilidade de leitos

Foram encontradas 45 citações que expressam a dificuldade do setor da regulação em garantir leitos aos usuários que se encontram nas urgências e emergências de hospitais, nas unidades de pronto atendimento (UPAS) ou nos domicílios, nos casos de procedimentos eletivos.

“Eu não digo a questão de mais hospitais, mas a oferta de leitos deve ser fidedigna. […] Um hospital que tenha 60 leitos, mas o que eles oferecem no dia são cinco, a gente sabe que não é verdade.” (G2)

“Tu estás visualizando os leitos vagos, e aí não tem como eles questionarem contigo, até porque os leitos são do SUS.” (G10)

“Uma mãe vem se queixando que precisa de uma determinada internação, e para mim é uma dificuldade quando vejo que não há vaga no sistema.” (G3)

“A regulação é fundamental para que a gente consiga atender um paciente que tá precisando e que esse paciente vá para o leito certo.” (PR9)

“[…] fazer atendimento telefônico na unidade, busca ativa de leitos por telefone, porque a gente faz supervisão no hospital, vai leito por leito…” (PR15)

“[…] e assim a carência de leito, querendo ou não, é absurda.” (PR4)

Grande parte das internações ocorre diretamente nos hospitais, sem intermediação de centrais de regulação; em algumas situações há um fluxo de encaminhamento diferenciado, com análise classificatória de prioridade/risco que por vezes revela problemas na acessibilidade, comprometendo a continuidade e a resolubilidade da atenção. O acesso aos serviços depende da localização da oferta e da moradia dos usuários, o que implica transporte, tempo, distância e custos de deslocamento.

Outro aspecto importante diz respeito à regulação dos leitos em hospitais privados conveniados ao SUS, que atendem planos de saúde e clientes particulares, os quais de certa forma competem com os usuários do sistema público de saúde na busca por leitos. O que se observa na prática é que os leitos cadastrados no SUS, são também utilizados pela rede privada para os pacientes de planos de saúde e particulares ^14^ .

No município de Belém, há profissionais de enfermagem que se revezam entre a central de internação e a supervisão nos hospitais na busca de leitos. Em outros municípios estudados, não há supervisão hospitalar de enfermeiros e a situação fica ainda mais difícil. Os hospitais públicos de maior complexidade, na maioria das vezes, estão com seus leitos ocupados, e os hospitais privados credenciados ao SUS precisam administrar seus leitos entre planos, convênios e particulares.

#### • Grande demanda

A grande demanda está representada em 68 citações. Há questões importantes que refletem a fragilidade no acesso de usuários do SUS, principalmente quanto às oportunidades de utilização dos serviços de saúde. As limitações de acesso apontam práticas de regulação pouco efetivas e dependentes de mecanismos não formais de ação, que repercutem em falta de vagas e de especialidades mais complexas ^15^ . Essas ideias são reforçadas pela atuação do poder judiciário, que interfere diretamente nos trâmites de encaminhamento das centrais de regulação. Os usuários descontentes, em razão da longa espera, procuram o Ministério Público (MP) ou a Defensoria Pública para fazer jus às suas demandas, conforme relatos:

“[…] uma demanda do Ministério Público, que tem aí uma outra urgência.” (G1)

“Toda semana a gente estava no MP, era bomba, era processo, é só crítica.” (G5)

“Nós recebemos demanda, mais MP, Defensoria e ouvidoria.” (G3)

“A demanda é crescente e a oferta é insuficiente.” (PR2)

“Não tem vaga pra todo mundo, o que deixa a gente meio angustiado.” (PR7)

“A promotora mandou, e ela queria saber o CID, queria saber da gravidade do paciente.” (PR8)

Nota-se que, na área da saúde, um dos objetos do processo judicial é a garantia de acesso ao atendimento, dificultado principalmente pela ausência de vagas. Esses pleitos judiciais demandados pela população podem indicar a necessidade de criação de políticas públicas voltadas para o rompimento das lógicas curativistas pautadas na medicalização e hospitalização, bem como o aparelhamento qualificado da atenção primária, o que amenizaria a busca pelo usuário à atenção terciária. Em razão disso, questiona-se a capacidade técnica dos representantes do poder judiciário para intervir na gestão do SUS ^16^ . É importante destacar que o aumento do número de idosos e da população ativa, sem a adequada inserção econômica, contribui para o aumento da violência, trazendo consequências marcantes em relação às respostas efetivas aos atendimentos de saúde ^17^ .

#### • Dificuldades sistêmicas em relação ao sistema de regulação (SISREG)

Encontram-se 40 citações vinculadas à operacionalização dos sistemas, especialmente o SISREG, e às relações com prestadores relacionadas às pactuações. Isso se expressa quando usuários necessitam de consultas e exames especializados que são usualmente autorizados na regulação, mas são barrados por cotas expiradas e precisam esperar até que sejam liberados novos períodos de solicitação e autorização.

“Nossas dificuldades são realmente as pactuações. A gente sabe da situação do país, do estado e do município; algumas coisas não estão sendo liberadas.” (G3)

“A regulação precisa de uma rede de atenção que trabalhe junto com ela.” (G7)

“Hoje a gente tem uma dificuldade com os laboratórios […].” (G8)

“Não conseguimos montar uma equipe que conheça como funciona o sistema de regulação.” (PR12)

“A questão é também descentralizar o SISREG para facilitar.” (PR16)

“A maioria dos municípios encaminha para Belém, onde tem a maior estrutura, a maior rede. A maioria desconhece PPI, regulação.” (PR12)

A implantação do SISREG tem minimizado os problemas sistêmicos, pois permite aos municípios o agendamento de consultas e exames especializados, assim como a solicitação de internações, de modo a viabilizar as demandas advindas da atenção primária, garantindo sua imparcialidade. Há de se convir que os processos de pactuação têm sido pouco satisfatórios e incondizentes com as reais necessidades de saúde da população, mesmo com o SISREG sendo executado.

O processo de pactuação de serviços, principalmente em relação aos municípios com baixa capacidade tecnológica, que demandam serviços de maior complexidade, acabam por sobrecarregar outros considerados de maior porte em termos de possibilidades de ofertar um maior leque de serviços à população. No entanto, isso não constitui uma regra, pois nos municípios de maior porte estão concentrados o maior número de serviços privados, que atendem planos de saúde e particulares, comprometendo por vezes a porta de entrada aos usuários do SUS.

É importante referir que o quantitativo de leitos daria para suprir a necessidade da população de municípios de grande porte, como Belém. No entanto, a pactuação permite que habitantes de municípios de menor resolutividade se desloquem para outros para dar continuidade às suas demandas de saúde. Sem a inserção de toda a capacidade hospitalar instalada numa central de regulação, há comprometimento da garantia da melhor resposta aos problemas de saúde dos usuários.

#### • Procedimentos de difícil agendamento e execução

Nessa questão estão inclusos os procedimentos de tomografia, ressonância magnética e os cirúrgicos de urologia e ortopedia. Em 20 citações, se reconhece que não há regulação de todos os procedimentos, principalmente quando os municípios não os realizam e necessitam buscar o atendimento em outros. No caso de pacientes de risco, tentam conceder-lhes prioridade.

“A questão do acesso à consulta especializada é um nó. O que deveria ser feito é um levantamento de possíveis pacientes de risco para dar uma prioridade a eles.” (G9)

“A gente vai esbarrar em outras dificuldades, como as tomografias de que no momento o município não dispõe.” (G11)

“Olha, eu acho que a maior dificuldade é justamente não conseguir fazer a regulação de todas as especialidades.” (PR14)

“[…] mas no caso da hemodiálise, quando vejo a fila muito grande, então a gente sabe que vaga é uma coisa limitada.” (PR17)

“A gente sabe que essa demanda só cresce e a gente precisa estar tentando ser o mais rápido possível para viabilizar consultas especializadas.” (PR18)

É crucial a ressignificação desses procedimentos e remodelagem do sistema assistencial para evitar o desperdício dos recursos e baixa resolutividade. Uma vez que a realização dos exames, no caso da ressonância nuclear magnética e tomografia computadorizada, ultrapassa o dobro do quantitativo preconizado nos parâmetros ministeriais, a rede de atenção secundária é sobrecarregada, sem esgotar o nível de atenção anterior. Nesse sentido, há necessidade de reordenação dos fluxos de acesso de forma articulada e solidária entre os entes federados

#### • Aumento da demanda reprimida de procedimentos eletivos

Este item foi evidenciado em 17 citações.

“A gente pega mais demanda cirúrgica de especialista” (G5)

“A gente está com uma demanda muito grande, não pode ofertar pra todos” (G3)

“[…] é a falta de leitos. Então nós temos uma demanda muito alta pra uma quantidade de leitos reduzidos, então que a gente tem que crivar, muitas vezes, não é justo” (PR7)

“A gente tem que saber quais as nossas prioridades, porque a gente tem uma demanda muito alta de pacientes eletivos.” (PR8)

O procedimento eletivo é todo atendimento prestado ao usuário em ambiente cirúrgico, com diagnóstico estabelecido e indicação de cirurgia a ser realizada com possibilidade de agendamento prévio, sem caráter de urgência ou emergência, devendo ocorrer no prazo máximo de 180 dias. Estudo revela que a demanda reprimida por cirurgia eletiva no país é causada principalmente pela defasagem na tabela de valores de procedimentos do SUS, que não cobre os reais valores do atendimento. O sacrificante tempo de espera de cirurgias eletivas constitui um problema de gestão em diferentes níveis de complexidade, tanto pela incapacidade em suprir a demanda como pela deficiente integração das redes de assistência ^18^ .

Vários aspectos reforçam que as centrais de regulação pouco contribuem para impactar o acesso de usuários aos procedimentos de média complexidade de responsabilidade municipal, atribuindo a esse descaminho a atuação centralizadora da gestão estadual, que dificulta muitas vezes a autonomia municipal ^19^ .

É importante observar que nos relatos, ora o processo regulatório deve ser mantido para garantir o atendimento, ora deve ser erradicado por atrapalhar os fluxos e burocratizar o sistema de saúde. A decisão precisará ser tomada e assumida para garantir o acesso do usuário aos serviços de saúde.

#### • Problemas no fluxo de informações entre a atenção primária e a regulação

Esse item foi encontrado 13 citações, como exemplificado a seguir:

“[…] isso acaba fazendo com que a central negue ou devolva a solicitação para que sejam acrescentadas novas informações.” (G6)

“A gente precisa ter esse conhecimento das ferramentas, dos sistemas que vão sendo trabalhados […]” (G9)

“Agora eu vejo uma carência muito grande na informação, tanto da parte do médico, do enfermeiro […]” (PR1)

“A questão da comunicação, do retorno imediato, nem que seja por telefone, por e-mail, a gente precisa de informações e estar sempre em comunicação com eles, e eles conosco.” (PR7)

A ausência de comunicação entre os serviços e/ou profissionais que compõem a atenção primária e a atenção especializada do município exercida nas centrais de regulação denota vulnerabilidade da rede assistencial ^20^ . A integração entre atenção primária e especializada deve ser encarada como uma medida estratégica para enfrentar diversos desafios do setor de regulação. Os desafios indiretos (nível mais político) podem ser enfrentados se a regulação apresentar consideráveis avanços nos desafios diretos (nível mais técnico). Isso vai exigir do setor habilidades no planejamento e execução de um plano estratégico para subsidiar o nível político com maior precisão ^21^ .

## Fatores Facilitadores da Regulação

As possibilidades mais presentes e significativas expressas nas narrativas dos gestores foram: ampliação da capacidade de conhecer a realidade do usuário do SUS, melhoria na atenção primária e incrementos de recursos financeiros para a saúde, capacitação e formação em saúde, além de reestruturação e reorganização dos procedimentos internos de regulação.

### • Ampliação da capacidade de conhecer a realidade do usuário do SUS

Essa necessidade encontra-se expressa em 10 narrativas.

“A regulação tem que conhecer toda a rede de extensão da saúde, ver de que forma vai oferecer os serviços aos nossos usuários a partir da demanda da atenção primária.” (G5)

“Eles têm várias patologias de base, eles são uma bombinha-relógio, de uma hora pra outra um enfarta, precisa internar, então a gente tem que tentar dar esse apoio.” (G10)

“[…] a gente teria como analisar melhor o real quadro clínico daquele paciente e nós teríamos como realmente triar da forma mais correta.” (PR14)

“A gente procura verificar o perfil do paciente pra estar encaminhando diretamente pro hospital que atenda às necessidades dele.” (PR18)

Os instrumentos da regulação pública como financiamento, rede prestadora de serviços, cadastros de unidades prestadoras, programação assistencial, centrais de regulação informatizadas e monitoramento das ações de atenção à saúde, entre outros, são importantes e constituem desafios na implementação da regulação pública Reforça-se a necessidade de ampliar os mecanismos regulatórios em razão de o SUS dispor de recursos limitados, sendo necessários critérios para priorização do acesso do usuário, em relação à oferta de ações e serviços de saúde proporcionais às diferentes necessidades ^22^ .

### • Melhoria na atenção primária e incrementos de recursos financeiros para a saúde

Esse tema recebe 57 citações, com relatos relacionados à falta de especialistas na rede de atenção primária, como também nas clínicas básicas de ginecologia e pediatria, à baixa resolutividade, que repercute em encaminhamentos considerados desnecessários à regulação, e à responsabilidade e compromisso em atender os usuários com uma resposta mais adequada aos seus problemas de saúde.

“A atenção básica tem o papel dela de atender o paciente, de estar mais perto, de ser mais responsável por ele.” (G2)

“Para melhorar o trabalho, deveriam melhorar a ponta.” (G4)

“A resolutividade tem que ser na unidade básica de saúde.” (G5)

“Eu acho que, se você melhorar a atenção básica, o paciente não vai chegar na especializada.” (G12)

“Se resolver a atenção básica, muita coisa vai melhorar.” (PR8)

“[…] porque a atenção básica daria conta de 90% dos casos.” (PR12)

“Deveria ter resolutividade na atenção primária.” (PR13)

“É importante que haja o fortalecimento da atenção básica porque, se não tiver, o fluxo não vai diminuir.” (PR14)

Os incrementos de recursos financeiros surgem nas narrativas em 33 citações:

“Eu acho que tem que ser dada mais atenção à parte financeira da saúde.” (G5)

“Pro serviço realmente ter uma melhora significativa, eu acho que precisa ter a rediscussão da programação pactuada integrada entre municípios” (G6)

“O sistema é único, todos os princípios e diretrizes bem-escritos, mas essa compensação financeira é necessária.” (PR14)

“Acho que se houvesse um recurso maior para contratar mais médicos, para reforçar a ponta […].” (PR17)

Entre as políticas ministeriais de incentivo à atenção primária, destaca-se o Programa de Melhoria do Acesso e da Qualidade da Atenção Básica (PMAQ-AB), cujo foco é atingir seu o objetivo com garantia de um padrão de qualidade, de maneira a permitir maior transparência e efetividade das ações de governo direcionadas a esse nível de atenção. Ele compreende quatro fases: adesão e contratualização, desenvolvimento, avaliação externa e recontratualização ^23^ . O PMAQ propõe em seus principais objetivos que haja mudanças nas práticas dos gestores e profissionais da atenção primária, para que ocorra a melhoria do acesso e da qualidade na rede de atenção à saúde ^24^ .

Um outro destaque se faz às redes de atenção à saúde, que se desenvolvem com base em um conjunto de elementos: o centro comunicador (atenção primária em saúde), os pontos de atenção secundários e terciários (serviços especializados), os sistemas de apoio (diagnósticos e terapêuticos, entre outros), os sistemas logísticos (cartão do usuário e outros) e o sistema de governança ^25^ .

As alternativas de reconstrução e valorização da atenção primária englobam: Programa de Requalificação das Unidades Básicas de Saúde, criação do novo Sistema de Informação da Atenção Básica (SIAB), Estratégia e-SUS Atenção Básica, Programa Telessaúde Brasil Redes, PMAQ-AB e Política Nacional de Atenção Básica, reestruturação do Programa Saúde na Escola, Programa Academia da Saúde, Política Nacional de Alimentação e Nutrição, Política Nacional de Educação Permanente em Saúde para os profissionais da atenção primária, Plano Nacional de Educação Médica, Programa de Valorização dos Profissionais na Atenção Básica e Programa Mais Médicos ^25^ . Convém ressaltar que várias mudanças estão ocorrendo após a ascensão do novo governo presidencial em 2019, e não se sabe ao certo a permanência das estratégias de reconstrução e valorização da atenção primária referidas acima.

### • Capacitação e formação em saúde

A necessidade da formação em saúde foi expressada em 56 citações.

“[…] porque existem várias situações que ocorrem aqui que às vezes a gente não tem uma coisa definida, às vezes fica muito no achismo, não existe algo escrito que me diga: ‘Você tem que utilizar essa ferramenta’.” (PR9)

“Eu acho que poderia ter mais treinamento, mais protocolos. Eu acho os protocolos fundamentais porque amparam o fazer do profissional.” (PR6)

“A gente tem que saber quais as nossas prioridades, porque a gente tem uma demanda muito alta.” (PR22)

“A gente tem que trabalhar multidisciplinarmente. […] Eu pergunto: ‘Olha, é paciente UTI? Quais são os resultados de exames? Tem exames? Tem tomografia? Tem ressonância?’” (PR20)

As instituições de saúde precisam implementar projetos de educação permanente, oportunamente articulados com instituições formadoras de recursos humanos. Tais projetos podem ocorrer no próprio espaço de trabalho, para desenvolver competências com foco nas melhorias e com a participação de todos os atores envolvidos, pautados na construção de conhecimentos de forma horizontal e interdisciplinar. Acima de tudo, a educação permanente em saúde permite formar profissionais na perspectiva do conceito ampliado de saúde, pautado nos princípios e diretrizes do SUS, com ênfase no controle social ^26^ .

### • Reestruturação e reorganização dos procedimentos internos de regulação

Esse item foi expresso em 14 citações.

“Tu não podes fazer nada fora sem ser organizado. A regulação até hoje eu vejo como o coração de toda uma gestão.” (G3)

“É acima de tudo garantir a estruturação da regulação, é possibilitar nessa estrutura o recebimento das demandas, tanto da capital quanto do interior, é descentralizar o acesso para consultas e exames pactuados.” (G8)

“Porque com a regulação a gente tem como controlar os leitos disponibilizados à rede pública, o atendimento ao usuário. Acho que é de fundamental importância na organização do serviço.” (G9)

O sucesso das centrais de regulação, que têm a pretensão de ser protagonistas no processo, depende de uma construção coletiva, e seus pontos de fortalecimento consistem no estabelecimento de parcerias com os prestadores

Em um estudo realizado em Diadema (SP), intitulado “De frente com os médicos: uma estratégia comunicativa de gestão para qualificar a regulação do acesso ambulatorial”, os médicos pontuam sua percepção enquanto reguladores e percebem a central de regulação ambulatorial como importante observatório da rede de atenção à saúde, capaz de produzir informações para subsidiar a tomada de decisão na gestão ^27^ . Há necessidade de reestruturação dos procedimentos de regulação para garantir acesso e equidade, e tornar a rede de saúde resolutiva e humanizada, constituindo uma potência de fomentar a avaliação para a tomada de decisão.

## CONCLUSÃO

O detalhamento dos fatores limitantes da regulação, expresso nos enunciados dos entrevistados, oferece uma panorâmica dos entraves enfrentados pelos gestores e profissionais reguladores da saúde. Demonstra a necessidade de levantar as vozes e unir forças em ações que contemplem a todos no acesso aos serviços de saúde. Essas ações devem se iniciar pela ampliação da capacidade em conhecer a realidade do usuário e pela implementação de políticas ministeriais que possibilitem a melhoria da atenção primária e a incrementação de recursos financeiros, que viabilizem a capacitação e a formação tendo como base a educação permanente em saúde e que permita a reestruturação e reorganização dos procedimentos internos de regulação, garantindo o acesso e a equidade dos serviços, apoiados em práticas resolutivas e humanizadoras em saúde.

Em resposta aos fatores limitantes, recomenda-se: a) melhoria na organização do fluxo assistencial em todos os níveis da rede de atenção à saúde, de modo a impactar nos indicadores de saúde mais sofríveis; b) garantia de leitos pela intermediação das centrais reguladoras da saúde; c) políticas que incentivem a desospitalização e assegurem a eficácia da atenção primária à saúde; d) qualificação dos trabalhadores na utilização dos diversos sistemas, dentre eles o SISREG; e) integração entre a atenção primária e a especializada.
